# Burnout and Exposure to Critical Incidents in a Cohort of Emergency Medical Services Workers from Minnesota

**DOI:** 10.5811/westjem.8.39034

**Published:** 2018-09-19

**Authors:** Lori L. Boland, Tyler G. Kinzy, Russell N. Myers, Karl M. Fernstrom, Jonathan W. Kamrud, Pamela J. Mink, Andrew C. Stevens

**Affiliations:** * Allina Health Emergency Medical Services, St. Paul, Minnesota; † Allina Health, Care Delivery Research, Minneapolis, Minnesota

## Abstract

**Introduction:**

Very little quantitative data on occupational burnout and exposure to critical incidents are available from contemporary United States emergency medical services (EMS) cohorts. Given that burnout has been associated positively with turnover intentions and absenteeism in EMS workers, studies that uncover correlates of burnout may be integral to combating growing concerns around retention in the profession.

**Methods:**

We administered a 167-item electronic survey that included the Maslach Burnout Inventory (MBI) and a modified version of the Critical Incident History Questionnaire (n=29 incident types) to paramedics, emergency medical technicians (EMTs), and dispatchers of a single ambulance service. We defined the presence of burnout as a high score on either the emotional exhaustion or depersonalization subscales of the MBI.

**Results:**

Survey respondents who provided regular 911 response at the time of the survey and completed the MBI portion of the survey were included in our analysis (190 paramedics/EMTs, 19 dispatchers; 54% response). The overall prevalence of burnout was 18%, with prevalence reaching 32% among dispatchers. The seven pediatric critical incident types presented in the survey accounted for seven of the top eight rated most difficult to cope with, and severity ratings for pediatric critical incidents did not differ by parental status (all p>0.30). A significant number of respondents reported that they had been threatened with a gun/weapon (43%) or assaulted by a patient (68%) at least once while on duty. Being over the age of 50, a parent, or in a committed relationship was associated with reduced odds of burnout in unadjusted models; however, these associations did not remain statistically significant in multivariate analysis. Increasing tertile of career exposure to critical incidents was not associated with burnout.

**Conclusion:**

Medical dispatchers may be an EMS subgroup particularly susceptible to burnout. These data also demonstrate quantitatively that in this EMS agency, responders find pediatric critical incidents especially distressing and that violence against responders is commonplace. In this study, a simple measure of career exposure to potentially critical incidents was not associated with burnout; however, individual reactions to incidents are heterogeneous, and assessment tools that more accurately enumerate encounters that result in distress are needed.

## INTRODUCTION

The physical and emotional toll of emergency medical services (EMS) work has been acknowledged for several decades,^1^–^4^ and likely contributes to turnover in the profession.^1^,^5^ Occupational stress in EMS is attributed to a number of factors including performance in potentially hostile or hazardous environments, repeated exposure to traumatic situations, the physical demands of the occupation, the strains of shift work, and the organizational and leadership stressors spawned by the hierarchical cultures prevalent in EMS.^2^,^6^

Occupational burnout has been documented extensively in emergency physicians^7^,^8^ and nurses 9 and has been linked to lower quality of care,^10^ but less is known about the prevalence and determinants of burnout in EMS clinicians, particularly those currently practicing in the United States (U.S.). With the exception of two recent reports,^11^,^12^ existing studies on burnout in U.S. EMS providers are more than two decades old.^3^,^4^,^13^,^14^ More recent studies from other parts of the world have examined burnout in EMS workers^6^, ^15^–^18^ using the Maslach Burnout Inventory (MBI), 19 the current gold standard for measurement of occupational burnout. Burnout has been associated positively with turnover intentions and absenteeism in cohorts of U.S. EMS workers;^11^,^12^ thus, empirical studies to uncover correlates of burnout may be integral to combating growing concerns around retention in the profession^20^ and optimizing quality and workforce engagement among EMS workers.

The potential for the development of post-traumatic stress symptoms in EMS personnel after exposure to critical incidents (CI) is well established,^6^, ^21^–^23^ and such exposures therefore likely influence provider wellbeing. However, research on the effects of CI exposure on emergency responders has largely focused on post-traumatic stress disorder (PTSD) or other clinically manifest symptoms (e.g., sleep disturbance), and have been conducted in relation to singular sentinel events such as mass casualty incidents or large-scale disasters. The scope and impact of cumulative exposure over the span of an EMS career to smaller scale events that are experienced more frequently but are still potentially disturbing has not been well described.

As part of a provider wellbeing initiative, we conducted a survey among the paramedics, emergency medical technicians (EMTs) and dispatchers in our ambulance service for the purposes of evaluating aspects of general mental wellbeing, informing refinement of support resources, and contributing to generalizable knowledge about mental wellbeing among EMS professionals. In addition to demographics, the survey included the MBI and a comprehensive inventory of exposure to CIs, which provided data about the career frequency and severity rankings for 29 CI types. The objectives of this study were to (1) determine the prevalence of burnout, (2) describe the relative career frequency and perceived severity of specific critical incident types, and (3) examine the association between burnout and a variety of provider factors, including demographics and cumulative exposure to CIs. We hypothesized that increasing cumulative exposure to CIs would be associated with increased levels of burnout.

**Population Health Research Capsule**

What do we already know about this issue?

Occupational burnout is common in emergency physicians and nurses, but little is known about the prevalence in emergency medical services (EMS) workers.

What was the research question?

What is the prevalence of burnout in our agency, what clinician factors are associated with burnout, and what critical incident types are perceived as most difficult?

What was the major finding of the study?

The overall prevalence of burnout in our agency was 18%, and reached 32% among dispatchers. Calls involving pediatric patients were rated most difficult.

How does this improve population health?

Reducing burnout in EMS workers may improve quality of care for patients and improve retention in the profession.

## METHODS

### Setting and Study Design

This cross-sectional survey was conducted at Allina Health EMS, a large ambulance service that provides 911 dispatch, advanced life support, basic life support and scheduled medical transport in approximately 100 communities in and around Minneapolis-St. Paul, Minnesota. The agency employs paramedics, emergency medical technicians (EMT), dispatchers, and support staff, and responds to just over 110,000 calls annually across a service area that covers 1,800 square miles. Crew configuration for all 911 responses in this system is indiscriminately paramedic-paramedic or paramedic-EMT; therefore, exposures and work environment are considered identical for the two certification classes and they have been analyzed in aggregate (hereafter paramedics).

In 2012, we emailed a 167-item electronic survey to all agency employees (n=479) regardless of role. The survey included assessments of occupational burnout and a variety of potential risk factors including demographics, social support, coping style and exposure to CIs. A penultimate draft was field-tested in a small number of paramedics employed by other ambulance agencies in the area who reported that the length and content was acceptable. Employees were told that the survey was voluntary and that there would be no individual follow-up. As an indirect incentive, each respondent was given the opportunity to designate one of three charities to receive a $10 donation on behalf of the ambulance service for their participation. The specific instruments used to assess burnout and exposure to CIs are described below. Additional details about the survey design and methods are available in “Supplemental Material.” The study protocol was approved by the Allina Health Institutional Review Board with voluntary completion of the survey constituting informed consent.

### Measures

We assessed occupational burnout using the 22-item MBI-Human Services Survey. 19 The MBI quantifies three dimensions of the burnout syndrome: emotional exhaustion (EE; 9 questions), depersonalization (DP; 5 questions) and reduced personal accomplishment (PA; 8 questions). Survey questions are stated as job-related feelings such as “*I feel emotionally drained from my work.*” Respondents indicate how often they feel this way with responses given on a scale from 0 (never) to 6 (every day), yielding the following ranges for the subscales: EE=0–54, DP=0–30, and PA=0–48. In addition to continuous subscale measures, we used previously described cutpoints based on normative U.S. data to define low, moderate, and high values on each scale (i.e., for EE, ≤16=low, 17–26=moderate, ≥27=high; for DP, ≤6=low, 7–12=moderate, ≥13=high; for PA, ≤31=low, 32–38=moderate, ≥39=high).^19^,^24^ Finally, a dichotomous construct was created, with burnout deemed present in those with a high score on the EE or DP subscale. This definition has been used by others,^25^–^28^ but approaches to using MBI subscales to determine the presence or absence of burnout are not consistent.^29^

We assessed exposure to CIs during EMS work using a modified version of the Critical Incident History Questionnaire (CIHQ).^30^ The CIHQ was initially developed for use with law enforcement officers, but similar to a previously described approach^23^,^31^ it was modified in this application by altering or removing items not relevant in EMS work. For example, “*Made a mistake in the line of duty that led to the serious injury or death of a fellow officer*” was replaced with “*Made a mistake that led to the injury/death of a patient.*” In addition, we added four pediatric incident types and items about mass casualty incidents, severe burn victims, and responding to incidents involving family/friends. The instrument also included two items related to violence against providers. The final instrument consisted of 29 CI types and indexed two dimensions of exposure – frequency and severity. For each incident type, the respondent was asked to estimate how many times during their career as a paramedic/dispatcher they had encountered that situation, using response categories of Never, 1, 2, 3,…9, 10–20, 21–50, or 50+. They were also asked to rate the severity of the incident type by answering the question “*In your opinion, how difficult would it be for paramedics/EMTs/dispatchers to cope with this type of incident?”* with ordinal responses ranging from 0 (*Not at all*) to 4 (*Extremely*).

The survey also contained basic demographic items including age, gender, current relationship status (single/not in a committed relationship, married/partnered), and parental status (yes, no). Respondents indicated their current position as Paramedic – Field staff, Paramedic – Supervisor/Manager, Dispatcher, Paramedic – Support staff (administration, education, clinical services etc.), interfacility transfer personnel, or other, with the first three categories used to identify the subset of respondents that provide regular 911 response. EMS tenure reflects the total number of years providing 911 response and/or direct patient care as a paramedic or dispatcher.

### Data Analysis

We summarized characteristics of the study participants and burnout measures using proportions (categorical variables) or means and standard deviations (continuous variables). Mean frequency and severity ratings for each of the 29 CI event types were computed and rank ordered to examine which event types were encountered most frequently and which were perceived as most difficult for providers. We examined crude prevalence of burnout across categories of a variety of provider characteristics, including age, gender, and EMS tenure. To examine cumulative career exposure to CIs as a risk factor for burnout, we summed the reported number of experienced incidents across all 29 event types for each respondent, with the response categories “10–20,” “21–50,” and “50+” assigned midpoint values of 15, 35.5, and 51, respectively. Tertiles of this measure of cumulative career frequency of CIs representing low, moderate, and high levels of exposure were then used in analysis. We used logistic regression to generate crude odds ratios of burnout in categories of provider characteristics and tertiles of cumulative CI exposure. Adjusted odds ratios were computed using multivariate logistic regression models that included all variables that had statistically significant univariate associations with burnout, i.e., age category, parental status, relationship status, provider role, and response setting. We performed all statistical analyses using Stata version 14.1 (StataCorp LP, College Station TX, USA).

## RESULTS

The overall survey response rate across all agency roles was 56% (266/479). We used human resources data to compare demographic characteristics of respondents with those of the target population where available, and the distributions of age, gender, years in current position and primary work setting among respondents closely reflected those of the agency as a whole.

At the time of the survey, 399 employees regularly provided 911 response, 217 of whom returned the survey (54% response). Among those 217, n=209 had complete data for the MBI and were used in this analysis. The average age in the analysis sample was 40, 60% were male, approximately two-thirds were parents, and 75% reported being married/partnered (Table 1). Slightly more than half reported they had been working in EMS for > 10 years, with nearly one third having an EMS tenure of 20+ years.

The overall prevalence of professional burnout in this cohort was 18% (Table 2). Using cutpoints derived from a normative U.S. sample, 6% and 15% of respondents scored high on the emotional exhaustion and depersonalization subscales, respectively, while 56% scored low on the dimension of personal accomplishment.

Survey respondents indicated that they perceived CIs involving children to be among the most difficult to experience and cope with. All seven of the pediatric incident types presented in the survey had very high average severity ratings, and accounted for seven of the top eight event types rated most difficult to cope with (Table 3). There was no difference in the mean severity ratings assigned by parents vs. non-parents for any of the seven pediatric incident types (all p>0.30). A strong inverse correlation of *r* = −0.72 (p<0.001) was observed between average severity rating and average reported career frequency across the 29 incident types. Using the median average severity rating (2.52) and the median average career frequency (3.92) to dichotomize incident types into high vs. low severity, and high vs. low frequency, four incident types emerged as being “high-frequency, high-severity” events: encountering a child that has been accidentally killed; encountering a child that has been severely injured; encountering a sudden infant death; and responding to a scene involving family/friends known to the crew. A significant number of respondents reported that they had been threatened with a gun/weapon (43%) or assaulted by a patient (68%) at least once while on duty during their EMS career.

The prevalence and odds ratios of burnout by provider characteristics and exposure to CIs are presented in Table 4. In univariate models, being over the age of 50, a parent, or in a committed relationship was associated with reduced odds of burnout. Dispatchers were at increased risk of burnout as compared to paramedics. This difference was not statistically significant, likely due to the small number of dispatchers in the analysis; however, the survey response rate among dispatchers was very high (76%; 19/25). There was no significant association between increasing tertile of cumulative career exposure to CIs, and burnout. Associations remained directionally consistent in a multivariate model, but none of the examined factors could be characterized as independently associated with burnout as all 95% confidence intervals included 1.0.

## DISCUSSION

### Burnout

Burnout has been linked to lower quality of care in other healthcare occupations;,^10^ therefore, understanding burnout and its correlates in EMS professionals may have implications for optimizing experience and outcomes for persons treated in the prehospital setting. The overall prevalence of burnout in this cohort was 18%, with particularly high levels of burnout occurring in dispatchers (32%), and in clinicians who did not have children (26%), or were not in a committed relationship (28%). Only 5% of providers over the age of 50 in our sample appeared to be experiencing burnout.

Two early studies that used the Burnout Scale for Health Professionals found burnout among EMS providers was more prevalent than in other healthcare professionals in the U.S.^3^,^4^ Two recent surveys conducted in U.S. paramedics and EMTs captured burnout measures using the Oldenburg Burnout Inventory and the Copenhagen Burnout Inventory.^11^,^12^ One reported a work-related burnout prevalence of 30% in paramedics and 19% in EMTs,^11^ and both found burnout was associated positively with turnover intentions and absenteeism.^11^,^12^ In the only prior report of MBI data from a cohort of U.S. paramedics, mean scores for EE, DP, and PA were 19.2, 9.3, and 28.1, respectively.^13^ MBI data from ambulance personnel outside the U.S. have been reported,^6^,^15^–^18^,^32^ but variability in defining burnout as a dichotomous construct makes inter-study comparisons difficult. Among Scottish ambulance personnel, the prevalence of high DP and high EE were 26% and 20%, respectively.^15^ Burnout among Dutch paramedics has been estimated at only 8.6%, but this prevalence is still higher than the 5.3% observed in a sample of the general working population in the Netherlands.^6^

Occupational burnout in large samples of employed physicians and the general working population of the U.S. has been estimated at 38% and 28%, respectively,^33^ both considerably higher than our observed overall prevalence of 18%. Recent MBI data from primary care physicians in our own health system revealed a burnout prevalence of 38%.^34^ While our paramedics appear to experience burnout at a comparatively low rate, the level of burnout among our dispatchers approaches the alarming level documented in physicians and exceeds that of the general working population of the U.S. Hypotheses about why burnout may be more prevalent among dispatchers in our agency include the high call volume and lack of “downtime” during shifts, stresses associated with operational accountability for a large number of crews and vehicles across an expansive coverage area, and the relatively sedentary environment. Dispatchers rarely have intervals void of incoming calls, whereas paramedics will often have some respite between patient encounters. To our knowledge, these are the first published data on dispatcher burnout, and studies in larger samples of this occupational subgroup are needed to elucidate whether this finding is unique to our agency.

### Critical Incidents

Symptoms of PTSD (e.g., intrusive memories, nightmares) occur in 10% of rescue workers worldwide, and estimates in EMS responders are consistently higher than those in firefighters and police officers.^35^ Logically, exposure to CIs has received a great deal of scrutiny as a primary contributor to the development of PTSD in rescue workers, with studies primarily focused on examining stress reactions after specific large-scale or widely-publicized events. But cumulative exposure to smaller-scale traumatic incidents outside the realm of extraordinary events may be equally deleterious, and examination of the full continuum of CI exposure in EMS workers is needed. The development of a comprehensive inventory to assess CI exposure in EMS professionals has been led by Donnelly and Bennett,^31^ who administered a modified version of the CIHQ in a sample of U.S. paramedics and EMTs. Their findings and suggested modifications served as the basis for the instrument used in our study.

Not unexpectedly, our data indicate that the most difficult CIs to cope with involve children, persons known to the crew, or a clinical error that results in an adverse outcome for a patient. A number of studies from around the world have presented paramedics and dispatchers with ad hoc lists of event types for severity ranking and comment.^4^,^6^,^23^,^31^,^32^,^36^,^40^ Consistent with our findings and irrespective of methods or geography, studies universally report that calls involving children or persons personally or professionally known to the crew are among the most disturbing. Unique to the current study, however, was an examination of incident severity rating by parental status. We hypothesized that emergency responders with children might find pediatric CIs more distressing because of mental and emotional transference of the situation to children in their own lives, but our findings did not support any difference in perceived severity by parental status.

Interpretation of frequency data from the modified CIHQ is less clear. We did not verify reported estimates of career frequencies as this was not feasible, so statements about absolute numbers of reported experiences would be speculative. However, similar to what has been observed in law enforcement officers,^30^ the total number of CIs experienced by each respondent was positively correlated with years in EMS (*r*=0.52; p<0.001), which offers some support for validity. The inverse correlation we observed between career frequency and severity rating (*r*= −0.72) is also comparable to that observed by Weiss et. al.^30^ in law enforcement officers (*r*= −0.61), and supports the hypothesis that frequent exposure to certain incident types may foster resilience.

Contrary to our hypothesis, we found no evidence that cumulative exposure to CIs in our responders is associated independently with professional burnout. This finding may be interpreted as being consistent with the viewpoint that an individual’s reactions after distressing incidents are of greater importance than the absolute number of potential CIs to which they are exposed. As noted by others, there is heterogeneity across individual emergency responders as to what constitutes a “critical incident,”^31^,^37^ and we readily acknowledge that the inventory used in this study only quantifies exposure to incident types with a high likelihood of heightened stress reactions and does not quantify the number of heightened reactions and resultant stress that is experienced. In the only other study that has attempted to quantify career exposure to CIs in EMS responders, the investigators observed that the correlation between lifetime CI exposure and a continuous measure of post-traumatic stress symptoms was relatively weak (*r*=0.25; p<0.01), and that more strongly correlated with post-traumatic stress symptoms was the level of stress that responders reported experiencing after such events (*r*=0.39; p<0.01).^31^ These findings suggest that a more ideal instrument for assessing cumulative CI exposure in EMS professionals would more strictly capture incidents that resulted in distress for the responder personally.

## LIMITATIONS

This study was conducted at a single, Midwestern EMS agency, and significant variation in EMS system models in terms of structure, volume, personnel attributes and geography likely compromise the generalizability of these results. Burnout may have been underestimated if employees who are disengaged were less likely to participate, or if those with extreme burnout have already exited the profession. However, providers who have strong concerns about work stress may have been more likely to embrace the opportunity to contribute to a wellbeing survey. Our response rate, while seemingly modest, is comparable with previous studies on the topic (40%–72%).^6^,^15^,^16^,^32^,^41^,^42^ We attempted to address the multifactorial nature of burnout by conducting multivariable analysis; however, our limited sample size resulted in wide confidence intervals and compromised our ability to make definitive statements about the predictive value of the factors examined.

### Implications

As a result of these findings, our agency instituted a process that offers timely chaplaincy support to providers after all potentially traumatic CIs, with particular attention to pediatric calls. Using real-time data mining, calls with specific trigger characteristics (e.g., pediatric death, more than four units on scene) generate an alert text message to the EMS chaplain who contacts the crew to offer support. A full-time EMS chaplain^43^ makes this protocol feasible, and the systematic approach acknowledges evidence that EMS providers are unlikely to seek assistance of their own volition after CIs.^44^,^45^ However, individualized response makes it difficult to accurately identify which calls will be troublesome^32^ and peer support models may be a more effective approach within existing EMS culture.^46^,^47^ We have also recently conducted paramedic focus groups to improve understanding of difficulties with pediatric calls. These initiatives represent an important starting point for both normalizing expression around stressors and altering the common perception among EMS providers that management is not concerned about their mental wellbeing and that agency support is inadequate.^15^–^17^,^40^,^47^

## CONCLUSION

Medical dispatchers in this sample exhibited a level of professional burnout commensurate with that of physicians and significantly higher than that experienced by the paramedics and EMTs who responded to the survey. These data also provide quantitative evidence that our EMS responders find pediatric CIs especially distressing, and that being threatened with a gun/weapon is commonplace in this population. In this study, a simple measure of career exposure to potentially critical incidents was not associated with burnout, but tools for more accurately capturing the number of incidents that resulted in distress are needed. EMS agencies should consider conducting assessments of burnout and other measures of wellbeing as a tool for mitigating systemic decline of wellbeing across the profession and averting personal tragedies in providers who are struggling.

## Supplementary Information



## Figures and Tables

**Table 1:** Characteristics of study population.

Variable	n = 209
Age, (years)	40 (12)
Age Category, (years)	
18–29	26% (55)
30–39	21% (43)
40–49	24 % (51)
50+	27% (56)
Not reported	2% (4)
Gender, % male	60% (125)
Parental status	
Parent	66% (137)
Not a parent	33% (69)
Not reported	1% (3)
Relationship status	
Married/Partnered	75% (157)
Single/Not committed	22% (46)
Not reported	3% (6)
EMS response role	
Paramedic	91% (190)
Dispatcher	9% (19)
EMS tenure (years)	
0–5	21% (43)
6–10	23% (49)
11–20	23% (49)
20+	33% (68)
Primary response setting	
Metro	70% (146)
Non-metro or rural	30% (62)
Not reported	< 1% (1)

*EMS*, emergency medical services.

Results are expressed as mean (SD) or percent (n).

**Table 2:** Burnout subscale measures and overall prevalence of burnout.

Variable	All Subjects (n=209)
MBI subscales	
Emotional exhaustion	
Mean (SD)	13.0 (8.6)
% Low	72%
% Moderate	22%
% High	6%
Depersonalization	
Mean (SD)	6.9 (5.9)
% Low	56%
% Moderate	29%
% High	15%
Personal accomplishment	
Mean (SD)	39.1 (6.2)
% Low	56%
% Moderate	33%
% High	11%
% with burnout	18% (37)

*MBI*, Maslach Burnout Inventory; *SD*, standard deviation.

**Table 3:** Rank-ordered mean severity ratings and mean reported career frequency of 29 critical incident types.

	Severity Rating Mean (SD)		Career Frequency Mean (SD)
Encountered a child that had been murdered	3.46 (1.0)	Encountered the body of someone recently dead	28.53 (19.6)
Encountered a child who had been badly beaten	3.25 (0.9)	Seen someone dying	26.48 (20.0)
Made a mistake that led to injury/death of a patient	3.20 (1.1)	Made a death notification	18.01 (19.3)
Encountered a child that had been accidentally killed	3.15 (0.9)	Encountered a suicide victim	14.80 (16.2)
Encountered a child that had been severely neglected	3.12 (1.0)	Encountered an adult who had been badly beaten	14.01 (16.4)
Encountered a child who had been sexually assaulted	2.99 (1.1)	Encountered a mutilated body or human remains	9.27 (14.4)
Encountered a SIDS death	2.93 (0.9)	Encountered a child that had been severely injured	8.86 (12.3)
Encountered a child that had been severely injured	2.75 (1.0)	Encountered an adult who had been sexually assaulted	7.11 (9.6)
Been present when coworker was seriously injured	2.74 (1.0)	Exposed to serious risk of AIDS/life-threatening diseases	6.64 (13.0)
Been threatened with a gun or other weapon	2.71 (1.0)	Encountered elderly person severely abused/neglected	5.87 (9.7)
Trapped in a potentially life-threatening situation	2.66 (1.1)	Encountered a SIDS death	4.81 (8.2)
Responded to a scene involving family/known to crew	2.63 (1.0)	Responded to a scene involving family/known to crew	4.75 (8.6)
Been seriously injured	2.62 (1.0)	Responded to a mass casualty incident	4.37 (8.0)
Been in a serious motor vehicle accident	2.58 (1.1)	Encountered a child that had been accidentally killed	4.24 (7.3)
Encountered elderly person severely abused/neglected	2.52 (0.9)	Exposed to life-threatening toxic substance	3.84 (10.7)
Had your life endangered in a large-scale disaster	2.50 (1.1)	Encountered a patient that was severely burned	3.92 (6.2)
Exposed to life-threatening toxic substance	2.33 (1.0)	Assaulted by a patient	3.56 (6.6)
Exposed to serious risk of AIDS/life-threatening diseases	2.30 (1.1)	Encountered a child that had been severely neglected	2.73 (6.0)
Encountered an adult who had been sexually assaulted	2.24 (1.0)	Encountered a child who had been sexually assaulted	2.49 (5.9)
Encountered a patient that was severely burned	2.23 (1.0)	Responded to a large-scale disaster	2.38 (5.3)
Responded to a large-scale disaster	2.17 (1.0)	Encountered a child who had been badly beaten	1.72 (3.0)
Encountered a mutilated body or human remains	2.16 (1.0)	Been threatened with a gun or other weapon	1.67 (3.8)
Encountered an adult who had been badly beaten	2.09 (1.0)	Trapped in a potentially life-threatening situation	1.40 (2.7)
Responded to a mass casualty incident	2.04 (1.1)	Been seriously injured	1.07 (3.3)
Made a death notification	1.99 (1.0)	Been present when coworker was seriously injured	0.81 (1.8)
Assaulted by a patient	1.99 (1.1)	Encountered a child that had been murdered	0.64 (1.8)
Encountered a suicide victim	1.96 (1.0)	Been in a serious motor vehicle accident	0.39 (0.9)
Seen someone dying	1.64 (1.0)	Had your life endangered in a large-scale disaster	0.34 (1.1)
Encountered the body of someone recently dead	1.45 (1.0)	Made a mistake that led to injury/death of a patient	0.23 (1.2)

*SD,* standard deviation; *SIDS*, sudden infant death syndrome; *AIDS*, acquired immune deficiency syndrome.

**Table 4:** Prevalence and odds ratios of burnout by provider characteristics and exposure to critical incidents.

Variable	Burnout	Unadjusted odds ratio (95% CI)	Adjusted^a^ odds ratio (95% CI)

	%		
Age category, (years)			
18–29	27%	1.00	1.00
30–39	21%	0.71 (0.27 – 1.82)	0.83 (0.27 – 2.53)
40–49	20%	0.65 (0.26 – 1.62)	0.98 (0.29 – 3.28)
50+	5%	0.15 (0.21 – 0.68)	0.27 (0.06 – 1.31)
Gender			
Male	18%	1.00	--
Female	18%	0.98 (0.48 – 2.03)	--
Parental status			
Parent	13%	1.00	1.00
Not a parent	26%	2.33 (1.12 – 4.85)	1.39 (0.49 – 3.95)
Relationship status			
Married/Partnered	15%	1.00	1.00
Single/Not committed	28%	2.30 (1.05 – 5.00)	1.46 (0.56 – 3.83)
EMS response role			
Paramedic	16%	1.00	1.00
Dispatcher	32%	2.37 (0.84 – 6.70)	2.15 (0.70 – 6.65)
EMS tenure (years)			
0–5	16%	1.00	--
6–10	27%	1.86 (0.66 – 5.19)	--
11–20	14%	0.86 (0.27 – 2.67)	--
20+	15%	0.89 (0.31 – 2.54)	--
Primary response setting			
Metro	21%	1.00	1.00
Non-metro or rural	10%	0.40 (0.16 – 1.01)	0.62 (0.23 – 1.68)
Tertile of critical incidents experienced during career			
Low (0 – 99)	13%	1.00	--
Moderate (100 – 226)	21%	1.82 (0.70 – 4.79)	--
High (> 226)	18%	1.49 (0.55 – 3.99)	--

*EMS*, emergency medical services; *CI*, confidence interval.

a Logistic regression model adjusted for age category, parental status, relationship status, response role and response setting.
