# Supplementary material for: Burnout and Exposure to Critical Incidents in a Cohort of Emergency Medical Services Workers from Minnesota
Source: West J Emerg Med. 2018 Sep 19;19(6):987–95. doi: 10.5811/westjem.8.39034 (PMC6225931; doi:10.5811/westjem.8.39034)
Supplement: Supplementary file 1 [file wjem-19-987-s001.doc]

**An assessment of mental wellbeing in a cohort of US emergency medical services workers in [*location*]: survey design and methods**

The following is a detailed description of a comprehensive cross-sectional survey that was conducted in the cohort of dispatchers, emergency medical technicians (EMTs) and paramedics at [*agency name*]. Using existing psychometric instruments, we designed and administered a survey to evaluate two measures of wellbeing – professional burnout and perceived stress – and an extensive list of potential risk factors, including demographics, social connectedness, coping style, and exposure to critical incidents.

***Conceptual Framework & Measured Constructs***

The conceptual framework for the survey is based in part on previous work by Sterud et al[^1^](#_ENREF_1) and is illustrated in **Figure 1**. The model proposes that a triad of individual factors, organizational factors, and critical incident exposure act in concert to influence the wellbeing of EMS workers. *Wellbeing* here refers to the level of self-perceived stress and occupational burnout present in each individual. Burnout and stress were assessed using the Maslach Burnout Inventory[^2^](#_ENREF_2) and Cohen’s Perceived Stress Scale[^3^](#_ENREF_3), which are described below. *Individual factors* are the unique aspects of personality, behavior, social networks and life experiences that each individual EMS clinician brings to the job (e.g. gender, marital status, level of social connectedness). *Agency factors* are the attributes of an EMS agency’s work environment and organizational culture (“chronic stressors”), whereas *critical incident exposure* refers to the degree to which an individual has witnessed or experienced traumatic events in the course of EMS work (“critical stressors”).

***Survey Instrument***

The survey comprises commonly-used, validated instruments that could be used to measure the desired constructs and risk factors in a relatively concise format. A penultimate draft of the 167-item instrument was field-tested in a small number of paramedics employed by other ambulance agencies in the area who reported that the length and content was acceptable.

*Occupational Burnout.* Burnout was measured using the Maslach Burnout Inventory – Health Services (MBI-HS) version[^2^](#_ENREF_2). This 22-item inventory is widely used for assessing work-related burnout through measurement of three dimensions: emotional exhaustion, depersonalization, and personal accomplishment. Survey items are stated as job-related feelings such as “*I feel emotionally drained from my work*” or “*I feel I’m positively influencing people’s lives through my work*”. Respondents indicate how often they feel this way about their job with responses given as a frequency ranging from 0 (never) to 6 (every day). High scores on either the emotional exhaustion or depersonalization subscales have been used to constitute burnout, but definitions are not consistent[^6^](#_ENREF_6).

*Perceived Stress.* Stress was assessed with the widely used 4-item version of Cohen’s Perceived Stress Scale (PSS)[^3^](#_ENREF_3). The 4 items measure how unpredictable, uncontrollable and overloaded the respondent perceives their life has been during the past month. For each item, respondents are asked how often they felt a certain way using a scale of 0 (*Never*) to 4 (*Very often*). Scores on the PSS range from 0 to 16, with higher scores indicating more perceived stress. The PSS has limited temporal reliability (i.e. respondents are only asked to reflect on feelings during the preceding month) and makes no distinction between stresses with a personal versus occupational origin. In contrast, MBI-measured burnout specifically targets work-related stress factors and reflects a more chronic state.

*Social Support.* Each respondent’s level of social integration was measured using the Berkman-Syme Social Network Index (BSNI)[^7^](#_ENREF_7). The BSNI is a validated 11-item questionnaire that yields a composite indicator of social support by assessing the nature of the respondent’s current social connections in four key areas: marital status, sociability (the number of close relatives and friends reported by the respondent and the frequency of contact with them), affiliation with a religious community or congregation, and membership in other organizations or clubs. The final score is used to characterize an individual’s level of social support as socially isolated, moderately isolated, moderately integrated, or socially integrated.

*Coping Style.* General coping responses were assessed using a combination of items from both the COPE Inventory[^8^](#_ENREF_8) and its shortened form, the Brief COPE[^9^](#_ENREF_9). Both can be used to ascertain how individuals tend to respond to stressful events in their lives. Statements such as “*I let my feelings out*” and “*I look for something good in what is happening*” are provided and respondents indicate for each the degree to which they usually exhibit the behavior or way of thinking when they are stressed. Responses are on a scale from 1 (*I usually do this a lot*) to 4 (*I usually don’t do this at all*) and are used to create subscales corresponding to a variety of coping styles such as “denial”, “venting and emotion”, and “active coping”. Two additional survey items previously used by others to form a “food coping” subscale[^10^](#_ENREF_10) were also included. While some of the coping styles are thought to be functional and others dysfunctional, the inventory is used strictly to describe the respondent’s general coping style and not to characterize that style as “positive” or “negative”.

*EMS Agency Factors.* Leiter and Maslach’s Areas of Worklife Survey (AWS)[^11^](#_ENREF_11) was used to measure the respondents’ perceptions of work environment. The AWS consists of 29-items that ascertain the degree of congruence between an individual and their job using subscales for each of the following six key areas: workload, control, rewards, community, fairness, and values. A series of 4-6 contrasting statements regarding organizational work environment are provided for each subscale (e.g. *“My values and the organization’s values are alike”* and *“Working here forces me to compromise my values*” are two statements for the *values* subscale), and respondents indicate their agreement with each statement using a 5-point scale ranging from 1 (s*trongly disagree*) to 5 (*strongly agree*). This validated and widely-used measure of job-person fit has been used in wellbeing assessments conducted in other provider groups in our health system.

*Critical Incident Exposure.* Exposure to critical events during the course of EMS work was assessed using a modified version of the Critical Incident History Questionnaire (CIHQ)[^12^](#_ENREF_12). Initially developed for use in law enforcement officers, the original CIHQ contains 34 types of critical incidents that can occur during the course of police work. The instrument indexes two dimensions of critical incident exposure – frequency and severity – by asking respondents to first indicate how often they encounter each type of incident and then to assign a severity rating that reflects the level of coping difficulty they associate with each incident type. Similar to an approach used by Donnelly et al, the CIHQ was modified in this application by altering or removing items from the original tool not relevant in EMS work. For example, “*Made a mistake in the line of duty that led to the serious injury or death of a fellow officer*” was replaced with “*Made a mistake that led to the injury/death of a patient*”, and “*Having to kill or seriously injure someone in the line of duty*” was removed. In addition, four specific pediatric incident types (e.g. “*Encountered a sudden infant death syndrome (SIDS) death*”) and items about mass casualty incidents, severe burn victims, and responding to incidents involving family/friends were added. The instrument also includes the following two items related to violence against EMS providers: “*Been threatened with a gun or other weapon*” and “*Been assaulted by a patie*nt”. The final survey consisted of 29 critical incident types. For each incident type, the respondent was asked to estimate how many times during their career as a paramedic/EMT/dispatcher they have encountered that situation, using response categories of Never, 1, 2, 3,…9, 10-20, 21-50, or 50+. They are then asked to rate the severity of the incident type by answering the question “*In your opinion, how difficult would it be for paramedics/EMTs/dispatchers to cope with this type of incident?”* with ordinal responses ranging from 0 (*Not at all*) to 4 (*Extremely*).

*Demographics and EMS Experience.* The survey also contained basic demographic items including age, gender, current relationship status (single/not in a committed relationship, married/partnered), parental status (yes, no), and highest education level (high school diploma or equivalent, some college, college graduate or beyond). Respondents indicated their current position with AHEMS as EMT/Paramedic – Field Staff, EMT/Paramedic – Supervisor/Manager, Dispatcher, EMT/Paramedic – Support staff (administration, education, clinical services), Interfacility transfer personnel, or other, with the first three categories used to identify the subset of respondents that provide regular 911 response. EMS tenure refers to the total number of years providing 911 response and/or direct patient care. Respondents also designated their primary work setting as either metropolitan or non-metro/rural. Per the agency’s coverage area at the time of the survey, metropolitan did not include any distinctly urban areas.

*EMS Chaplain Resource.* Since 2007, [*agency name*] has employed a dedicated chaplain whose primary role is to build relationships with and offer support to the agency’s EMS responders. Five questions were included at the end of the survey to gather information about awareness, perception, and use of the in-house EMS chaplain resource.

***Survey Administration***

In September 2012, an electronic link to the web-based survey was distributed via email to all [*agency name*] employees regardless of role (n=479). During the three weeks prior to survey distribution, employees received multiple electronic communications from senior leaders and the EMS chaplain about the nature and objectives of survey, and flyers announcing the survey were posted in all ambulance bases across the system. In these communications, potential respondents were told that the survey was voluntary, it would take approximately 20 minutes to complete, responses would remain confidential and there would be no individual follow-up as a result of the survey. They were also informed that at the end of the survey, there would be an opportunity for them to designate one of three local charities to receive a $10 donation on behalf of the ambulance service as indirect compensation for their participation in the survey. Respondents were encouraged to contact the EMS chaplain if any questions or concerns arose as a result of completing the survey (e.g. intrusive memories). The study protocol was approved by the [*name of designated IRB*] Institutional Review Board, with voluntary completion of the survey constituting informed consent.

**References**

1. Sterud T, Ekeberg O, Hem E. Health status in the ambulance services: a systematic review. *Bmc Health Serv Res*. 2006;6:82.

2. Maslach C, Jackson SE, Leiter MP. Maslach Burnout Inventory Manual (3rd ed) Palo Alto, CA: Consulting Psychologists Press; 1996.

3. Cohen S, Kamarck T, Mermelstein R. A global measure of perceived stress. *J Health Soc Behav*. 1983;24:385-96.

4. Dyrbye LN, Varkey P, Boone SL, Satele DV, Sloan JA, Shanafelt TD. Physician satisfaction and burnout at different career stages. *Mayo Clin Proc*. 2013;88:1358-67.

5. Thomas NK. Resident burnout. *JAMA*. 2004;292:2880-9.

6. Dyrbye LN, West CP, Shanafelt TD. Defining burnout as a dichotomous variable. *J Gen Intern Med*. 2009;24:440; author reply 1.

7. Berkman LF, Syme SL. Social networks, host resistance, and mortality: a nine-year follow-up study of Alameda County residents. *Am J Epidemiol*. 1979;109:186-204.

8. Carver CS, Scheier MF, Weintraub JK. Assessing coping strategies: a theoretically based approach. *J Pers Soc Psychol*. 1989;56:267-83.

9. Carver CS. You want to measure coping but your protocol's too long: consider the brief COPE. *Int J Behav Med*. 1997;4:92-100.

10. Tsenkova V, Boylan JM, Ryff C. Stress eating and health. Findings from MIDUS, a national study of US adults. *Appetite*. 2013;69:151-5.

11. Leiter MP MC. Areas of Worklife Scale manual. 4th ed. ed. Wolfville, NS: Centre for Organizational Research Development, Acadia University2006.

12. Weiss DS, Brunet A, Best SR, Metzler TJ, Liberman A, Pole N, et al. Frequency and severity approaches to indexing exposure to trauma: the Critical Incident History Questionnaire for police officers. *J Trauma Stress*. 2010;23:734-43.

13. Donnelly E. Work-related stress and posttraumatic stress in emergency medical services. *Prehosp Emerg Care*. 2012;16:76-85.

14. Donnelly EAB, Bennett M. Development of a critical incident stress inventory for the emergency medical services. *Traumatology*. 2014;20:1-8.

| **Supplementary Data** |  |  |  |  |
| --- | --- | --- | --- | --- |
| Characteristics of target population and survey respondents | | | | |
|  |  |  |  |  |
|  | **Survey Respondents** | |  | **Target Population (n = 479)** |
| **Variable** | 911 Responders (n = 217) | ALL (n = 266) |  |
|  |  |  |  |  |
| Age, *y* | 39.8 (11.5) | 40.6 (12.0) |  | 39.0 (12.4) |
|  |  |  |  |  |
| Age ≥ 50 yrs | 26.9% (57) | 29.7% (77) |  | 32.2% (154) |
|  |  |  |  |  |
| Gender |  |  |  |  |
| Male | 60.9% (131) | 63.6% (168) |  | 67.0% (321) |
| Female | 39.1% (84) | 36.4% (96) |  | 33.0% (158) |
|  |  |  |  |  |
| Years in current position | 9.4 (9.5) | 8.7 (9.1) |  | 7.9 (8.3) |
|  |  |  |  |  |
| Primary work setting |  |  |  |  |
| Metro | 70.4% (152) | 74.2% (193) |  | 75.3% (345) |
| Non-Metro or Rural | 29.6% (64) | 25.8% (67) |  | 24.7% (113) |
|  |  |  |  |  |
| Results are expressed as mean (SD) or percent (n) | | | | |
